# Correlation of Fibrinogen-Albumin Ratio With Gensini Score in ST-Segment Elevation Myocardial Infarction

**DOI:** 10.7759/cureus.88317

**Published:** 2025-07-19

**Authors:** Abhijit Hanamantaraya Utnal, Siddanagouda Biradar, Anuja Kadagud, Shreyas N, Manoj Kumar

**Affiliations:** 1 Internal Medicine, BLDE (Deemed to be University) Shri B. M. Patil Medical College, Hospital and Research Centre, Vijayapura, IND; 2 General Medicine, BLDE (Deemed to be University) Shri B. M. Patil Medical College, Hospital and Research Centre, Vijayapura, IND

**Keywords:** coronary artery disease, fibrinogen-albumin ratio, gensini score, inflammatory biomarkers, stemi

## Abstract

Introduction

ST-segment elevation myocardial infarction (STEMI) is a severe form of coronary artery disease (CAD) often associated with systemic inflammation. The fibrinogen-albumin ratio (FAR) has been proposed as a novel inflammatory marker with potential prognostic value. The Gensini score, derived from coronary angiography, quantifies the severity of CAD. This study aimed to assess the correlation between FAR and Gensini score in STEMI patients.

Materials and methods

A cross-sectional observational study was conducted at a tertiary care center in Karnataka, India, from May 2023 to December 2024. Seventy patients with a clinical diagnosis of STEMI were enrolled. Clinical evaluation, blood tests (including fibrinogen and albumin levels), echocardiography, and coronary angiography were performed. FAR was calculated by dividing fibrinogen (g/L) by albumin (g/L), and Gensini scores were computed to assess CAD severity. Statistical analysis included Pearson's correlation and the chi-squared test.

Results

The study population comprised mostly men (47, 67.14%) and individuals aged over 60 years (40, 57.14%). Single and double vessel disease were each present in 24 (34.3%) patients. FAR >0.1 was seen in 37 (52.86%). A significant association was found between Gensini score and the extent of vessel disease (p=0.0001); however, FAR did not show a statistically significant correlation with Gensini score (r=-0.002; p=0.989). FAR also lacked a significant association with heart failure types.

Conclusion

FAR may have limited utility in predicting angiographic severity in patients with STEMI. While it remains a simple and economical inflammatory marker, its prognostic relevance in acute coronary events appears uncertain. Further research with larger cohorts is warranted to validate its clinical applicability.

## Introduction

Coronary artery disease (CAD) remains a leading cause of morbidity and mortality worldwide [1]. Among its various manifestations, acute ST-segment elevation myocardial infarction (STEMI) represents the most severe and life-threatening form. STEMI is characterized by a distinct electrocardiographic pattern reflecting complete occlusion of a coronary artery [1,2]. Although advances in preventive strategies and healthcare delivery have led to a decline in CAD incidence in developed countries, developing nations continue to witness a rising trend in myocardial infarction (MI) due to lifestyle transitions, limited access to healthcare, and underutilization of preventive interventions. The first-year mortality following STEMI is still substantial, estimated at approximately 10%, despite the widespread availability of timely revascularization techniques such as percutaneous coronary intervention (PCI), coronary artery bypass grafting (CABG), and dedicated cardiac critical care services [3,4]. Moreover, emerging evidence highlights that social determinants, such as socioeconomic status, education, and neighborhood environment, are increasingly recognized as pivotal contributors to cardiovascular disease risk and clinical outcomes, further complicating the global burden of ischemic heart disease [4].

Fibrinogen, a key acute-phase reactant, plays an essential role in systemic inflammation and blood viscosity regulation. Elevated fibrinogen levels have been associated with an increased risk of thrombosis and have been recognized as independent predictors of CAD and MI [5]. Furthermore, fibrinogen enhances the expression of various pro-inflammatory cytokines, contributing to a pro-thrombotic and pro-inflammatory milieu within the coronary vasculature [6]. These properties position fibrinogen as a potential biomarker reflecting both thrombotic risk and inflammatory status in acute coronary syndromes [7]. Conversely, serum albumin is a negative acute-phase reactant with anti-inflammatory properties. Hypoalbuminemia has been implicated in impaired endothelial function, increased oxidative stress, and heightened coronary vasoreactivity [8]. These effects are partly attributed to elevated levels of free lysophosphatidylcholine, which alter blood rheology and promote vasoconstriction. Numerous studies have identified an inverse relationship between serum albumin levels and the severity of CAD, reinforcing the role of albumin in modulating cardiovascular risk [9,10].

The Gensini scoring system is a widely accepted angiographic tool for quantifying the severity of CAD. It takes into account the anatomical location, degree of luminal narrowing, and extent of lesions in the coronary vasculature. By providing a structured method for evaluating coronary atherosclerosis, the Gensini score aids clinicians in stratifying patient risk, guiding therapeutic interventions, and predicting clinical outcomes [11]. Integration of laboratory biomarkers such as the fibrinogen-albumin ratio (FAR) with angiographic scoring may offer an enhanced, non-invasive approach to evaluating CAD severity, particularly in the context of acute STEMI [12]. FAR values were derived from routine admission blood tests (fibrinogen and albumin), which are typically available within 1-2 hours of patient presentation, well before cardiac catheterization [13]. Given this background, the present study aimed to calculate the FAR in patients presenting with STEMI and to determine its association with the severity of CAD, as assessed by the Gensini score following coronary angiography. Establishing such a correlation may help incorporate FAR as a convenient and cost-effective adjunct marker in the risk stratification and early detection of CAD severity in acute MI.

## Materials and methods

This cross-sectional observational study was conducted over a period of one and a half years, from May 2023 to December 2024, at BLDE (Deemed to be University) Shri B. M. Patil Medical College, Hospital and Research Centre, Vijayapura, Karnataka, India. The study population included patients admitted to the intensive coronary care unit (ICCU) and emergency wards with a diagnosis of STEMI, based on inclusion and exclusion criteria. A total of 70 patients were enrolled after obtaining written informed consent and ethical clearance from the Institutional Ethics Committee of BLDE (Deemed to be University) (approval number: BLDE(DU)/IEC/929/2023-24).

All patients were initially diagnosed with STEMI using the criteria outlined in the 2018 "Fourth Universal Definition of Myocardial Infarction" (ESC/ACC/AHA/WHF). STEMI was confirmed by the presence of a new ST-segment elevation at the J-point in at least two contiguous leads, with the following cut-offs: ≥1 mm in all leads other than V2 and V3 and for leads V2 and V3, ≥2 mm in men ≥40 years, ≥2.5 mm in men <40 years, and ≥1.5 mm in women regardless of age.

Detailed clinical evaluation included demographic details (age, gender), history of comorbidities (hypertension, diabetes mellitus, thyroid disorders), medication use, and personal habits (smoking, alcohol). Physical examination covered general and systemic parameters, including blood pressure, pulse rate, jugular venous pressure, height, weight, and body mass index (BMI). A baseline 12-lead electrocardiogram (ECG) was recorded for all patients to detect ST-T changes, followed by laboratory investigations including complete blood count, random blood glucose, serum creatinine, serum albumin, and serum fibrinogen. The FAR was calculated as fibrinogen (g/L) divided by albumin (g/L).

Two-dimensional echocardiography was performed for the assessment of regional wall motion abnormalities (RWMA) and left ventricular ejection fraction (LVEF). All eligible patients underwent coronary angiography followed by PCI where indicated. The Gensini score was used to quantify the severity of CAD, calculated from angiographic data by assigning weighted scores based on the degree and location of stenosis in the coronary arteries.

Sample size estimation was based on a correlation coefficient (r) of -0.38 between the FAR and the Gensini score, as reported by Charach et al. [14]. At a 95% confidence level (α=0.05) and 90% power (β=0.10), the sample size was calculated using the formula $N=\left(\frac{Z_{\alpha}+Z_{\beta}}{C}\right)^2+3$ where Zα=1.9600, Zβ=1.0364, and C=0.5×ln((1+r)/(1-r))=0.4001. Substituting the values and rounding up for adequacy and to accommodate potential dropouts, the final sample size was taken as 70 participants.

Data were entered into Microsoft Excel (Microsoft Corporation, Redmond, Washington, United States) and analyzed using IBM SPSS Statistics for Windows, Version 26.0 (Released 2019; IBM Corp., Armonk, New York, United States). Categorical variables were compared using the chi-squared test, while Pearson's correlation coefficient was used to evaluate the relationship between FAR and Gensini score. A p-value of <0.05 was considered statistically significant.

## Results

The majority of patients were above 60 years of age, accounting for 40 (57.14%), followed by 28 (40%) in the 40-60-year group and two (2.86%) below 40 years. Men constituted 47 (67.14%) and women 23 (32.86%) of the study population. Based on BMI classification, 25 (35.7%) had normal weight, 22 (31.42%) were classified as obese I, 13 (18.6%) as overweight, seven (10%) as obese II, and three (4.28%) as underweight (Table 1).

**Table 1:** Baseline demographic and clinical characteristics of the study participants (n=70) Frequency and percentages were calculated for all variables. BMI: body mass index

Parameter	Category	Frequency	Percentage (%)
Age group (years)	<40	2	2.86%
	40-60	28	40%
	>60	40	57.14%
Sex	Male	47	67.14%
	Female	23	32.86%
BMI	Underweight (<18.5)	3	4.28%
	Normal (18.5-22.9)	25	35.7%
	Overweight (23-24.9)	13	18.6%
	Obese I (25-29.9)	22	31.42%
	Obese II (≥30)	7	10%

Hypertension was present in 23 (32.86%) participants, while diabetes mellitus was reported in 21 (30%) and a combination of both in nine (12.86%). Tobacco and smoking were each reported in 17 (24.28%) subjects, alcohol use in seven (10%), overweight status in 13 (18.57%), and obesity in 29 (41.43%). Anterior wall MI was the most frequent type seen in 38 (54.28%), followed by inferior wall in 26 (37.14%), posterior wall in five (7.14%), and lateral wall in one (1.44%) (Table 2).

**Table 2:** Risk factors and infarction characteristics (n=70) Frequency and percentages were calculated for all variables. MI: myocardial infarction

Parameter	Category	Frequency	Percentage (%)
Risk factors	Hypertension	23	32.86%
	Diabetes mellitus	21	30%
	Diabetes+hypertension	9	12.86%
	Smoking	17	24.28%
	Tobacco	17	24.28%
	Alcohol	7	10%
	Overweight	13	18.57%
	Obesity	29	41.43%
Location of MI	Anterior wall	38	54.28%
	Inferior wall	26	37.14%
	Lateral wall	1	1.44%
	Posterior wall	5	7.14%

Single vessel disease (SVD) and double vessel disease (DVD) were equally prevalent, seen in 24 (34.3%) each, followed by triple vessel disease (TVD) in 20 (28.6%) and recanalized vessels in two (2.8%). Among the heart failure types, heart failure with reduced ejection fraction (HFrEF) (<40%) was observed in 41 (58.6%), heart failure with mildly reduced ejection fraction (HFmrEF) (41-50%) in 21 (30%), and heart failure with preserved ejection fraction (HFpEF) (>50%) in eight (11.4%) (Table 3).

**Table 3:** Distribution of coronary artery disease and heart failure types (n=70) Frequency and percentages were calculated for all variables. SVD: single vessel disease; DVD: double vessel disease; TVD: triple vessel disease; HFrEF: heart failure with reduced ejection fraction (EF <40%); HFmrEF: heart failure with mildly reduced ejection fraction (EF 41-50%); HFpEF: heart failure with preserved ejection fraction (EF >50%)

Parameter	Category	Frequency	Percentage (%)
Coronary artery disease	SVD	24	34.3%
	DVD	24	34.3%
	TVD	20	28.6%
	Recanalized	2	2.8%
Heart failure type	HFrEF (<40%)	41	58.6%
	HFmrEF (41-50%)	21	30%
	HFpEF (>50%)	8	11.4%

**Figure 1:**
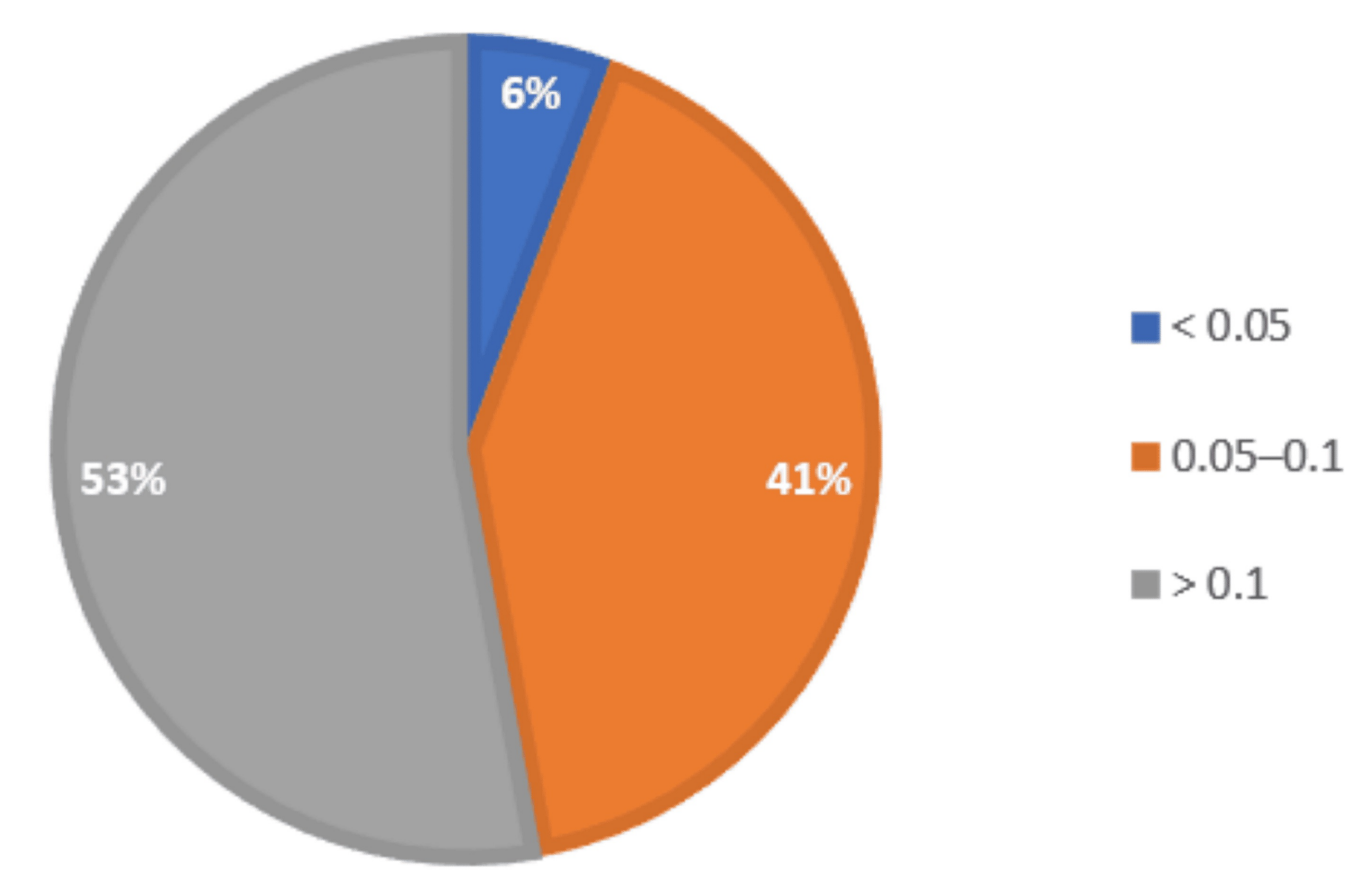
Pie chart showing FAR FAR: fibrinogen-albumin ratio

FAR levels were >0.1 in 37 (52.86%), 0.05-0.1 in 29 (41.42%), and <0.05 in four (5.72%) (Figure 1).

Patients with mild Gensini scores (<30) mostly had HFpEF (13, 50%), while moderate scores were predominantly associated with HFpEF (18, 66.7%). Severe scores (>60) were most frequently associated with HFrEF (six, 35.3%). However, the association between Gensini score categories and heart failure types was not statistically significant (χ²=3.1662; df=4; p=0.5304) (Table 4).

**Table 4:** Correlation between Gensini score and heart failure (n=70) Frequencies were calculated for all variables, and the chi-square test was applied to assess associations. A p-value of less than 0.05 was considered statistically significant. Chi-square (χ²)=3.1662; df=4; p=0.5304 HFrEF: heart failure with reduced ejection fraction (EF <40%); HFmrEF: heart failure with mildly reduced ejection fraction (EF 41-50%); HFpEF: heart failure with preserved ejection fraction (EF >50%)

Gensini score	HFrEF	HFmrEF	HFpEF	Total
Mild (<30)	8 (30.77%)	5 (19.23%)	13 (50%)	26
Moderate (30-60)	7 (25.93%)	2 (7.41%)	18 (66.67%)	27
Severe (>60)	6 (35.29%)	1 (5.88%)	10 (58.82%)	17

An increasing severity in Gensini scores was significantly associated with more extensive vessel involvement. Among patients with SVD, most had mild scores (15, 62.5%), whereas in TVD, severe scores were most common (11, 55%). This association was statistically significant (χ²=26.3804; df=6; p<0.0001) (Table 5).

**Table 5:** Correlation between Gensini score and vessel involvement (n=70) Frequencies were calculated for all variables, and the chi-squared test was applied to assess associations. A p-value of less than 0.05 was considered statistically significant. Chi-square (χ²)=26.3804; df=6; p<0.0001 SVD: single vessel disease; DVD: double vessel disease; TVD: triple vessel disease

No. of vessels	Mild	Moderate	Severe	Total
SVD	15 (62.5%)	6 (25%)	3 (12.5%)	24
DVD	8 (33.33%)	13 (54.17%)	3 (12.5%)	24
TVD	1 (5%)	8 (40%)	11 (55%)	20
Recanalized	2 (100%)	0 (0%)	0 (0%)	2

Among patients with FAR >0.1, 23 (62.16%) had HFrEF, and six (16.22%) had HFpEF. In those with FAR 0.05-0.1, HFrEF was seen in 15 (51.72%), and in the FAR <0.05 group, three (75%) had HFrEF. However, the association between FAR categories and heart failure types was not statistically significant (χ²=7.6916; df=4; p=0.1035) (Table 6).

**Table 6:** Correlation between FAR and heart failure (n=70) Frequencies were calculated for all variables, and the chi-squared test was applied to assess associations. A p-value of less than 0.05 was considered statistically significant. Chi-square (χ²)=7.6916; df=4; p=0.1035 FAR: fibrinogen-albumin ratio; HFrEF: heart failure with reduced ejection fraction (EF <40%); HFmrEF: heart failure with mildly reduced ejection fraction (EF 41-50%); HFpEF: heart failure with preserved ejection fraction (EF >50%)

FAR range	HFmrEF	HFpEF	HFrEF	Total
<0.05	0 (0%)	1 (25%)	3 (75%)	4
0.05-0.1	13 (44.83%)	1 (3.45%)	15 (51.72%)	29
>0.1	8 (21.62%)	6 (16.22%)	23 (62.16%)	37

There was no statistically significant correlation between fibrinogen (r=-0.032; p=0.790), albumin (r=-0.164; p=0.174), or FAR (r=-0.002; p=0.989) with Gensini score (Table 7).

**Table 7:** Correlation coefficients between Gensini score and serum parameters (n=70) Pearson's correlation coefficient was used to evaluate the relationship with Gensini score. A p-value of <0.05 was considered statistically significant. FAR: fibrinogen-albumin ratio

Parameter	Correlation coefficient (r)	P-value
Fibrinogen	-0.032	0.790
Albumin	-0.164	0.174
FAR	-0.002	0.989

## Discussion

CAD is a chronic inflammatory disorder, with inflammation playing a pivotal role across all stages of atherosclerosis. Among the various inflammatory biomarkers, plasma fibrinogen has been extensively studied and recognized as an independent risk factor for CAD in population-based studies such as the ARIC trial. However, its additional predictive value in healthy individuals, when compared to traditional risk factors, remains modest [5]. Albumin, on the other hand, is a negative acute-phase reactant with anti-inflammatory properties. During acute ischemic events like STEMI, hypoalbuminemia has been associated with increased reperfusion injury and adverse outcomes [15,16]. FAR, which integrates the effects of both markers, has emerged as a potential non-invasive biomarker for identifying patients at increased cardiovascular risk [17].

STEMI management has evolved significantly, especially in high-resource settings where timely primary PCI has become the standard of care [18]. In contrast, low-resource settings face delays due to limited PCI access, which makes early biomarker-based diagnosis critically important. In the present study, conducted among 70 STEMI patients, most were aged over 60 years (40, 57.14%), and men constituted 47 (67.1%) of the population. These findings are consistent with the study by Karahan et al., where the mean age was 61.5 years and men made up 203 (73%) of the sample [19]. The burden of traditional risk factors was high, with smoking and tobacco use seen in 34 (48.56%), hypertension in 23 (32.86%), diabetes in 21 (30%), and obesity in 29 (41.43%) patients, trends that are broadly consistent with previous Indian data on STEMI, including that reported by Rasool et al. [20].

Anterior wall MI was the most common type in our study (38, 54.28%), followed by inferior wall MI (26, 37.14%). Similar patterns were observed by Sharma et al., who reported anterior wall MI in 117 (58.2%) and inferior wall MI in 84 (41.8%) patients [21]. Our angiographic findings revealed SVD in 24 (34.3%) and multivessel disease (DVD and TVD) in 44 (62.9%), differing slightly from Sharma et al.'s study, where SVD predominated [21]. These differences may be due to variations in referral patterns, regional risk profiles, or healthcare access.

When assessing the correlation between FAR and the severity of CAD, as measured by the Gensini score, our results did not show a statistically significant relationship (r=-0.002; p=0.989). A similar lack of association was noted between FAR and different types of heart failure. While other studies have indicated a potential role of FAR in predicting adverse cardiovascular outcomes [22,23], the absence of correlation in our data may reflect limitations such as the relatively small sample size and single-center design. Furthermore, as a laboratory-based parameter, FAR is vulnerable to pre-analytical and analytical variations that could affect its reliability.

This study has certain limitations that merit consideration. First, the sample size was relatively small (n=70). Second, the single-center design may affect the generalizability of the findings to broader populations with different demographic and clinical profiles. Third, FAR levels may be influenced by various confounding factors such as nutritional status, acute-phase reactants, liver function, and hydration status, which were not controlled for in our analysis. Additionally, serial measurements of FAR were not performed, which could have provided more dynamic insights into its clinical relevance over time.

Strengths of the study include the evaluation of FAR as a simple, non-invasive, and rapid laboratory test that could be especially useful in patients refusing invasive coronary angiography. It provides a convenient, cost-effective adjunct for early CAD risk assessment in resource-limited settings and may aid in the triaging of acute coronary syndrome cases.

## Conclusions

This study found no significant correlation between the FAR and angiographic severity of CAD in STEMI patients, as measured by the Gensini score. In contrast, the Gensini score showed a strong association with the extent of vessel involvement, reaffirming its clinical utility. Unlike prior studies suggesting prognostic value of FAR, our findings suggest it may not reliably reflect CAD burden or outcomes in acute STEMI. This divergence may be due to differences in study population, inflammatory status, or methodology. Despite its simplicity and accessibility, the role of FAR in acute coronary syndromes remains uncertain. Further large-scale, multicenter studies are warranted to evaluate its prognostic potential and long-term clinical relevance.
